# The association between hearing impairment and polymorphisms of genes encoding inflammatory mediators in Japanese aged population

**DOI:** 10.1186/s12979-014-0018-4

**Published:** 2014-11-26

**Authors:** Yasue Uchida, Saiko Sugiura, Hiromi Ueda, Tsutomu Nakashima, Fujiko Ando, Hiroshi Shimokata

**Affiliations:** Department of Otorhinolaryngology, Aichi Medical University, Nagakute, Aichi prefecture Japan; Department of Otorhinolaryngology, National Center for Geriatrics and Gerontology, Obu City, Aichi prefecture Japan; Department of Otorhinolaryngology Cognitive and Speech Medicine, Nagoya University School of Medicine, Nagoya City, Aichi prefecture Japan; Department of Health and Medical Sciences, Aichi Shukutoku University, Aichi prefecture, Japan; Department for Development of Preventive Medicine, National Center for Geriatrics and Gerontology, Obu City, Aichi prefecture Japan; Graduate School of Nutritional Sciences, Nagoya University of Arts and Sciences, Nisshin, Japan

**Keywords:** Age-related hearing loss, Polymorphism, Longitudinal study, TNFα

## Abstract

**Background:**

Aging process is accompanied by a chronic sub-clinical systemic inflammation. This study aimed to assess the association between hearing impairment and polymorphisms of genes encoding cytokines deeply-committed to the inflammatory response and immune homeostasis in an elderly Japanese population. Data were collected in the Longitudinal Study of Aging surveyed biennially between 1997 and 2010. The participants without any missing information at baseline were 1,957 individuals, and the gross accumulated number of 8,675 subjects (40–89 years of age) was analyzed. Two hearing impairment criteria were taken as the better ear pure-tone average (PTABE) greater than 25 dB and greater than 40 dB. We analyzed cumulative data using generalized estimating equations to investigate the effect of 9 polymorphisms, namely, tumor necrosis factor (*TNF*) α, rs1800630; TNF receptor super family (*TNFRSF*) 1B, rs1061624; interleukin *(IL)-1A*, rs1800587; *IL-1B*, rs16944; *IL-4R*, rs1801275; *IL-6*, rs1800796; *IL-10*, rs1800872; *IL-1* receptor-associated kinase 1 (*IRAK1*), rs1059702; C reactive protein (*CRP*), rs1130864.

**Results:**

The odds ratios for the hearing impairment (PTABE >25 dB) risk under additive genetic model were significant in *TNF-*α rs1800630 and *TNFRSF1B* rs1061624, which were respectively 1.172 (confidence interval [CI]: 1.005-1.367), 1.211 (CI: 1.053-1.392) in model after adjustment for possible confounders. Using the criterion of PTABE >40 dB as disabling hearing impairment, the association remains significant in *TNFRSF1B* rs1061624, but not in *TNF-*α rs1800630. No other polymorphisms showed a significant association.

**Conclusions:**

The present population-based cohort study demonstrated that *TNF-*α rs1800630 and *TNFRSF1B* rs1061624 contributed to the incremental risk of hearing impairment in the elderly. TNF-α and TNF receptor interactions play a pivotal role in the pathogenesis of the inflammatory response, and also cause programmed cell death and cell proliferation. The present observation implied the signalling cascades of TNF were involved in ear aging.

## Background

Chronic low-grade inflammation is the hallmark of the aging process and is now accepted as a fundamental pathogenic mechanism in the development of several age-related degeneration including cardiovascular disease [1], atherosclerosis [2], cognitive disorders [3], and type 2 diabetes [4].

The low-grade proinflammatory status appearing during the aging process has become termed ‘inflammaging’, coined by Franceschi et al. [5]. Physiological aging is characterized by elevated levels of inflammatory mediators. Serum pro-inflammatory cytokines such as interleukin(IL)-6 and tumor necrosis factor (TNF) α and acute phase proteins such as C-reactive protein (CRP) show an increase with age [5]. Further, the levels of cytokines that counteract the inflammatory state, such as IL-10, are reduced with age [6] compounding the inability to maintain immune homeostasis.

The subjects in the present analyses were derived from the National Institute for Longevity Sciences - Longitudinal Study of Aging (NILS-LSA), a population-based biennial survey of a cohort of approximately 2,200 community dwellers. In the present study, we intended to clarify the relationship between hearing impairment and nine polymorphisms of genes encoding inflammatory mediators, namely, *TNF-*α C-863A, rs1800630; TNF receptor superfamily member (*TNFRSF*) 1B G593A, rs1061624; *IL-1A* -889C/T, rs1800587; *IL-1B* -511C/T, rs16944; *IL-4R* G1902A, rs1801275; *IL-6* C-572G, also known as C-634G, rs1800796; *IL-10* A-592C, rs1800872; IL-1 receptor-associated kinase 1 (*IRAK1*) T587C, rs1059702; *CRP* +1444 C > T, rs1130864. Our ultimate aim is to get closer to the pathophysiological elucidation of presbycusis as an age-related disease.

## Results and discussion

Table 1 shows the demographic profiles of the study subjects at baseline and cumulative data. Of the 1957 participants at baseline, the mean age was 59.2 years, and rates of male and hearing impairment with the World Health Organization (WHO) criteria were 50.4% and 20.1%, respectively. Allelic and genotypic distribution of 9 single-nucleotide polymorphisms (SNPs) in subjects for the present analyses at baseline are indicated in Table 2.

**Table 1:** **Demographics of study subjects**

	**Baseline (1st wave)**	**Cumulative (1st-6th wave)**
Number	1957	8675
Sex, % male	50.4	51.5
Mean age (SD), year	59.2 (10.9)	62.0 (10.4)
Average hearing threshold for frequencies 0.5,1, 2, 4 kHz for the better ear (SD), dB	17.3 (12.0)	19.7 (12.1)
Hearing impairment with the WHO criteria, %	20.1	25.0
Ear disease history, %	27.4	30.2
Occupational noise exposure, %	22.8	21.4
Hypertension, %	24.9	29.6
Diabetes, %	7.5	8.2
Dyslipidemia, %	16.7	19.7
Stroke, %	2.6	3.8
Cardiac disease, %	11.8	13.4
Smoking, %	44.2	43.2

**Table 2:** **Allelic and genotypic distribution of subjects for the present analyses at baseline**

**Gene**	**rs No**	**Allele** ^**1)**^	**Allele N= 3914**	**Number (%)**	**Genotype**	**N= 1957**	**Number (%)**
Tumor necrosis factor-α (TNF-α)	rs1800630	A (C)	C	A	CC	CA	AA
			3229 (82.5)	685 (17.5)	1420 (72.5)	389 (19.9)	148 (7.6)
Tumor necrosis factor receptor superfamily member 1B (TNFRSF1B)	rs1061624	A (G)	G	A	GG	GA	AA
			1916 (49.0)	1998 (51.0)	473 (24.2)	970 (49.6)	514 (26.2)
Interleukin-1A (IL1A)	rs1800587	T (C)	C	T	CC	CT	TT
			3525 (90.1)	389 (9.9)	1587 (81.1)	351 (17.9)	19 (1.0)
Interleukin-1B (IL1B)	rs16944	T (C)	C	T	CC	CT	TT
			2094 (53.5)	1820 (46.5)	546 (27.9)	1002 (51.2)	409 (20.9)
Interleukin 4 receptor (IL-4R)	rs1801275	G (A)	A	G	AA	AG	GG
			3375 (86.2)	539 (13.8)	1454 (74.3)	467 (23.9)	36 (1.8)
Interleukin-6 (IL-6)	rs1800796	G (C)	C	G	CC	CG	GG
			2969 (75.9)	945 (24.1)	1134 (58.0)	701 (35.8)	122 (6.2)
Interleukin-10 (IL-10)	rs1800872	C (A)	A	C	AA	AC	CC
			2648 (67.7)	1266 (32.3)	896 (45.8)	856 (43.7)	205 (10.5)
Interleukin 1 receptor-associated kinase-1 (IRAK-1)	rs1059702	C (T)	T	C	TT	TC	CC
			3000 (76.6)	914 (23.4)	1312 (67.0)	376 (19.2)	269 (13.8)
C-reactive protein 2 (CRP2)	rs1130864	T (C)	C	T	CC	CT	TT
			3670 (93.8)	244 (6.2)	1721 (87.9)	228 (11.7)	8 (0.4)

^1)^Reference alleles are indicated in parentheses.

The results of multivariable analysis using logistic regression with generalized estimating equations (GEEs) were demonstrated in Table 3. Of the 9 SNPs analyzed, there were significant associations of polymorphic alleles in *TNF-*α and *TNFRSF1B* with risk increments for hearing impairment, based on the criterion of PTABE >25 dB. The odds ratios (ORs) for hearing impairment (PTABE >25 dB) risk in *TNF-*α rs1800630 were 1.120 (95% confidence interval [CI]: 0.979-1.281) in model 1, 1.192 (CI: 1.020-1.392) in model 2, and 1.172 (CI: 1.005-1.367) in model 3. The ORs in *TNFRSF1B* rs1061624 were 1.151 (CI: 1.021-1.297) in model 1, 1.210 (CI: 1.052-1.393) in model 2, and 1.211 (CI: 1.053-1.392) in model 3. The significance was still observed even after adjustment for possible confounders in model 3. Using the criterion of PTABE >40 dB as disabling hearing impairment, the association between polymorphic allele and hearing impairment remain significant in *TNFRSF1B* rs1061624, but not in *TNF-*α rs1800630. The ORs for hearing impairment (PTABE >40 dB) risk in *TNFRSF1B* rs1061624 were 1.272 (CI: 1.052-1.538) in model 1, 1.293 (CI: 1.045-1.599) in model 2, and 1.275 (CI: 1.026-1.584) in model 3.

**Table 3:** **Hearing impairment risk of inflammatory mediators related SNPs analyzed using logistic regression with generalized estimating equations**

**Gene**	**rs No**	**Crude: model 1**			**Adjusted: model 2**			**Adjusted: model 3**		
		**Odds ration**	**95% CI**	**p value**	**Odds ration**	**95% CI**	**p value**	**Odds ration**	**95% CI**	**p value**
*TNF-*α	rs1800630									
	Hearing impairment criteria									
	PTABE ^1)^>25 dB	1.120	0.979-1.281	0.100	1.192	1.020-1.392	0.027	1.172	1.005-1.367	0.044
	PTABE 40 dB	1.090	0.885-1.344	0.417	1.109	0.884-1.390	0.372	1.106	0.884-1.384	0.380
*TNFRSF1B*	rs1061624									
	PTABE >25 dB	1.151	1.021-1.297	0.022	1.210	1.052-1.393	0.008	1.211	1.053-1.392	0.007
	PTABE >40 dB	1.272	1.052-1.538	0.013	1.293	1.045-1.599	0.018	1.275	1.026-1.584	0.028
*IL1A*	rs1800587									
	PTABE >25 dB	1.093	0.894-1.335	0.386	1.061	0.845-1.331	0.611	1.070	0.855-1.339	0.557
	PTABE >40 dB	0.975	0.692-1.373	0.885	0.946	0.660-1.354	0.760	0.956	0.667-1.371	0.807
*IL1B*	rs16944									
	PTABE >25 dB	0.941	0.834-1.060	0.316	0.915	0.796-1.052	0.212	0.920	0.801-1.057	0.238
	PTABE >40 dB	0.924	0.762-1.121	0.422	0.960	0.774-1.190	0.706	0.945	0.760-1.174	0.608
*IL-4*	Rrs1801275									
	PTABE >25 dB	0.986	0.829-172	0.872	1.038	0.851-1.265	0.715	1.031	0.847-1.255	0.761
	PTABE >40 dB	0.968	0.729-1.85	0.822	1.010	0.746-1.367	0.949	1.050	0.774-1.424	0.756
*IL-6*	rs1800796									
	PTABE >25 dB	1.011	0.881-1.161	0.872	1.083	0.924-1.269	0.327	1.079	0.922-1.262	0.343
	PTABE >40 dB	1.034	0.829-1.289	0.770	1.042	0.822-1.320	0.734	1.056	0.833-1.339	0.651
*IL-10*	rs1800872									
	PTABE >25 dB	0.981	0.864-1.114	0.764	0.983	0.844-1.145	0.826	0.973	0.838-1.131	0.725
	PTABE >40 dB	0.887	0.714-1.102	0.280	0.860	0.572-1.101	0.231	0.834	0.651-1.070	0.153
*IRAK-1*	rs1059702									
	PTABE >25 dB	1.026	0.912-1.55	0.666	1.012	0.889-1.151	0.862	1.010	0.888-1.148	0.885
	PTABE >40 dB	0.887	0.718-1.097	0.268	0.8161	0.707-1.051	0.141	0.869	0.713-1.058	0.162
*CRP2*	rs1130864									
	PTABE >25 dB	0.976	0.758-1.257	0.81	1.099	0.816-1.479	0.535	1.092	0.817-1.461	0.552
	PTABE >40 dB	0.924	0.624-1.363	0.692	1.081	0.711-1.643	0.715	1.113	0.726-1.705	0.623

^1)^PTABE : pure-tone average threshold at 0.5, 1, 2 and 4 kHz for the better ear.

Moderating variables.

model 1 : No variables.

model 2 : Age and sex.

model 3 : Any history of noise exposure, ear disease, hypertension, diabetes, dyslipidemia, stroke and cardiac disease, and lifetime smoking habit in addition to those of model 2.

In the other 7 SNPs, no significant risks of hearing impairment in either criterion were observed in models with different combination of moderator variables.

In the present study, we assessed the contribution of polymorphic genes encoding inflammatory mediators to hearing loss in an elderly Japanese community-dwelling population. The results suggested that *TNF-*α rs1800630 and *TNFRSF1B* rs1061624 may be involved in risk increment of age-related hearing impairment. The significant ORs observed both in two hearing impairment criteria implied a substantial impact of *TNFRSF1B* rs1061624 on the susceptibility of age-related hearing impairment, since hearing impairment of the better ear means bilateral impairment and thus WHO defines disabling hearing impairment.

TNF-α is a proinflammatory cytokine that is a master regulator of vascular proatherogenic changes and the pathogenesis of inflammatory diseases [7]. It serves a function in the regulation of cell differentiation, proliferation and apoptosis, also in inflammation, innate and adaptive immune responses. TNF-α does not work independently at the cellular level. It activates intracellular signaling cascades via binding to two types of receptors, TNF receptor superfamily member 1A (TNFRSF1A/ also known as p55 andTNFR1) and member 1B (TNFRSF1B/ also known as p75 and TNFR2), then exerts its pleiotropic role. TNFRSF1A is expressed on most human cell types, while TNFRSF1B expression is restricted to certain subpopulations of specific neuronal subtypes, endothelial cells, and cells of the immune system [8,9].

Many researchers have substantiated emergence of TNF-α and other mediators in the inflammatory response of inner ear. TNF-α is secreted mainly by activated macrophages, monocytes, T cells, B cells, and fibroblasts [10]. In the cochlea, it is reported that spiral ligament fibrocytes have been shown to release chemokines including TNF-α when challenged with otitis media pathogens [10]. Other authors have reported the role of inflammatory cytokines, particularly TNF-α, in inner ear inflammation [11–13]. Studies using immunohistological and genetic amplification analyses from University of Debrecen in Hungary reported that elevated TNF-α receptor expression correlates with histologic activity of otosclerosis [14]. Also they reported that histologic otosclerosis exhibits a strong correlation with measles virus presence in the bone and related inflammation with TNF-α and speculated that the TNF-α chronically released from the foci enters the inner ear fluid spaces in histologically active stages of otosclerosis and may cause outer hair cell functional disorder and subsequent sensorineural hearing loss [15].

Recently inflammatory responses in inner ear under various damaging conditions including noise-overstimulation have been reported [16,17]. Fujioka, et al. evaluated the time-dependent expression of proinflammatory cytokines in noise-exposed rat cochlea, and suggested that proinflammatory cytokines, including TNF-α, IL-1 β, and IL-6 might initiate an inflammatory response and could have some role in the mechanism of noise-induced cochlear damage. Hwang, et al. evaluated tinnitus and mRNA expression levels of *TNF-*α, *IL-1*β, and N-methyl D-aspartate receptor subunit 2B (*NR2B*) genes in cochlea and inferior colliculus of mice after intraperitoneal injections of salicylate, then concluded that salicylate treatment resulting in tinnitus augmented expression of the *TNF-*α, *IL-1* β genes in cochlea and inferior colliculus [18].

One study in experimental animals demonstrated that the use of a TNF-α-neutralizing agent could prevent the sensorineural hearing loss secondary to induced pneumococcal meningitis [19]. Meanwhile, the potential cytotoxicity of TNF-α, itself on cochlear sensory cells is open to debate. Keithley, et al. tested for circulating leukocytes recruitment induced by TNF-α in the guinea pig inner ear and examined hair cells in culture directly exposed to TNF-α [20]. TNF-α infused into the guinea pig cochlear scala tympani in the absence of antigen or pathogens resulted in infiltration of leukocytes around the venules and within scala tympani. In their interpretation, TNF-α is sufficient to recruit inflammatory cells to the cochlea. However, there was no associated hearing loss as measured with a click stimulus. And TNF-α at concentrations used in the in-vivo experiments applied directly to organ of Corti explants caused minimal hair cell death. They concluded that the profound hearing loss and severe loss of inner hair cells observed in antigen-induced labyrinthitis cannot be explained by direct TNF-α mediated cytotoxicity alone.

Through extensive examinations of expression and function, some genetic variations have been shown to explain inter-individual variation. SNPs in the *TNF-*α, *TNFRSF1A* and *TNFRSF1B* genes have been identified, however functional data pertaining to these polymorphisms in scarce. Rs1800630 is a common functional polymorphism, involving a C to A substitution at position −863. It is located in the proximal promoter of the *TNF-*α gene. The presence of DNA sequence variations in regulatory region might interfere with transcription of *TNF-*α gene, and with increase in the susceptibility to human wide range of diseases (infectious, cancer, autoimmune, neurodegenerative and other diseases). Qidwai & Khan reviewed *TNF-*α gene polymorphism and disease prevalence comprehensively [7]. In the review, several mechanisms playing important role in gene regulation were discussed. Two most important factors that interfere with the gene regulation are promoter hypermethylation and the presence of polymorphism in regulatory region, and their hypothesis was that during disease conditions, there was upregulation or downregulation of gene (transcriptional dysregulation). *TNF-*α polymorphism rs1800630 A-allele was associated with lower specific anti-pneumococcal IgG levels compared with children carrying C⁄C genotype of rs1800630, in one instance [7]. In the present analyses, significant odds ratios for hearing impairment risk were obtained even after adjustment for possible influential variables. The minor A-allele of rs1800630 polymorphism increased the risk of hearing impairment.

There are limited data regarding the functionality of the various SNPs in the *TNFRSF1B* gene. One clinical cancer investigation demonstrated that the *TNFRSF1B* rs1061624 genotype was a predictive factor of clinical response i.e., a complete response or not, to treatment with a 5-fluorouracil (5-FU)/cisplatin (CDDP) - based chemoradiotherapy in Japanese esophageal squamous cell carcinoma patients [21]. Carrying the rs1061624 variant was reported to affect the phenotype of inflammatory bowel diseases in a New Zealand Caucasian population (decreased the risk of ulcerative colitis in the left colon) [22]. In our results, *TNFRSF1B* rs1061624 was associated with risk increment of age-related hearing impairment defined as a hearing level of PTABE >25 dB and PTABE >40 dB.

Recently, we found a significant association of risks for both sudden sensorineural hearing loss (SSNHL) and Ménière’s disease with *IL-1A* rs1800587 polymorphism [23], and also reported that carriers of the *IL-6* rs1800796 minor-allele were potentially more susceptible to SSNHL than noncarriers [24]. From one Korean case–control investigation, the presence of the *IL-4R* polymorphism Q576R, also known as G1902A, rs1801275 [25], was reported to be a risk factor for SSNHL [26]. In the present results, however, no associations of the presence of the 7 SNPs other than *TNF-*α rs1800630 and *TNFRSF1B* rs1061624, were found with the risk of age-related hearing impairment.

Several potential limitations of the present study should be cited. The genes in the selection of the present analyses were highly polymorphic respectively. To cite an example, 32 SNPs lies in transcription factor–binding site of 20 transcription factors have been detected in the *TNF-*α upstream region [7]. But restrictions in budget and efficiency allowed us only to investigate widely and shallowly. Selection criterion of SNPs in the NILS-LSA study was based on candidate polymorphisms analysis for aging or geriatric diseases, such as senescence, geriatric syndrome, dementia, and osteoporosis. We focused the association between candidate SNPs for aging and hearing, then previously published the effects on hearing in the elderly, of *EDN1*, *MTHFR*, *MTR* (folate metabolism), *UCP1*, *UCP2*, and *FABP2*, oxidative stress related SNPs [27–30].

In addition, we were not able to obtain each inflammatory cytokine level in individuals. However, recent experimental observation, which explored the cochlear cytokine expression pattern under normoxia and hypoxia, suggested presence of tissue-specific regulatory pathways even among three different cochlear tissues; the organ of Corti, stria vascularis together with spiral ligament, and modiolus [31]. Given the tissue-specificity of cochlear tissue, even if blood cytokine level during common condition is obtained, it cannot be directly linked to pathogenetic elucidation. Further basic and applied researches will be required to interpret the role of each SNP in hearing deterioration.

From the aspect of methodology, there may be some other way to define a criterion for hearing impairment by different age groups, such as comparison with normal hearing levels according to the International Organization for Standardization (ISO) standard 7029:2000 [32]. ISO-7029 is based on a linear model of the hearing threshold for populations of otologically normal persons within the age limits of 18 years to 70 years, not including the older ages. Because the age range of the present subjects was distributed up to 89 years of age, we did not use a hearing assessment according to the ISO-7029.

The strength of this study is found in a feature of the NILS-LSA subject group. The NILS–LSA cohort consists of inhabitants who were selected randomly from resident registrations and stratified according to both age and sex. The risk of genotypic bias in subjects was therefore likely to be minimal. There were no significant differences in genotype frequencies of *TNF-*α rs1800630 and *TNFRSF1B* rs1061624 between the HapMap-JPT values and those obtained for the current participants. The ancestral allele of the rs1061624 single nucleotide variation is A-allele. The reference allele in the present analyses was G-allele, and G-allele frequencies of the rs1061624 polymorphism were released in the frequency reports as 0.535 in HapMap-JPT (Japanese in Tokyo), 0.539 in 1000 Genomes Project JPT (Japanese in Tokyo), 0.390 in AoD_Japanese, 0.507 in JBIC-allele (752 anonymous unrelated Japanese volunteers), and 0.580 in JPT_GENO_PANEL.

## Conclusion

The present population-based cohort study demonstrated that *TNF-*α rs1800630 and *TNFRSF1B* rs1061624 contributed to the incremental risk of hearing impairment in the elderly Japanese population. TNF-α and TNF receptor interactions play a pivotal role in the pathogenesis of the inflammatory response, and also cause programmed cell death and cell proliferation. The present observation implied that the signaling cascades of TNF-α were involved in age-related hearing loss.

## Materials and methods

### Subjects

The NILS-LSA is a comprehensive and interdisciplinary study to observe age-related changes and consists of various gerontological and geriatric measurements, such as medical examinations, blood chemical analysis, body composition, anthropometry, physical function, nutritional analysis, psychological tests, and visual and auditory function. The participants were community-dwelling adults of Aichi Prefecture in central Japan, who were randomly selected from resident registrations, stratified by both age and sex in cooperation with the local government. The numbers of men and women recruited were similar, and age at baseline was 40 years to 79 years, with similar numbers of participants in each decade of age (40s, 50s, 60s, and 70s). Most of medical check-up are examined repeatedly every two years, and venous blood samples for genetic test were collected only at the first-wave examination. Sera and DNA samples were stored in deep freezers for later examination. The study protocol was approved by the Committee of Ethics of Human Research of the National Center for Geriatrics and Gerontology (#14, #52, #74). Written informed consent was obtained from all participants. The details of the NILS-LSA have been described elsewhere [30,33].

Data were collected from the NILS-LSA participants who partook at least in the first-wave examination. Those who did not consent to have blood samples taken, and those who lacked results for any of the nine SNP genotype analyses were excluded. The participants at baseline were 2,149 adults, and the cumulative data were 9,358 samples in accumulated total, which were compiled from the first through the sixth-wave examination surveyed between 1997 and 2010. Participants filled out a series of questionnaires in advance of the examination visit. Ear disease history and experience of occupational noise exposure were taken account as basic informations in an evaluation of age-related hearing impairment. Histories of hypertension, diabetes, dyslipidemia, stroke, and cardiac disease were also taken from the self-reported account. Lifetime smoking history was categorized as never or ever. Those who had a deficiency of any required information for the present analyses and those who did not complete the hearing test were excluded. The subjects for the present analyses without any missing information were 1,957 individuals at baseline, and the gross accumulation number of subjects, regardless of repetitive visits, was 8,675 adults 40 years to 89 years of age. The mean number of repetitive visits was 4.4.

### Genetic analyses and other measures

Genomic DNA was extracted from peripheral blood lymphocytes using the standard procedure and polymerase chain reaction (PCR) amplification was performed. Nine polymorphisms of genes encoding inflammatory mediators, namely, *TNF-*α C-863A, rs1800630; TNF receptor superfamily member (*TNFRSF*) *1B* G593A, rs1061624; *IL-1A* -889C/T, rs1800587; *IL-1B* -511C/T, rs16944; *IL-4R* G1902A, rs1801275; *IL-6* C-572G, also known as C-634G, rs1800796; *IL-10* A-592C, rs1800872; IL-1 receptor-associated kinase 1 (*IRAK1*) T587C, rs1059702; *CRP* +1444 C > T, rs1130864 were investigated.

Genotyping was carried out using a fluorescence method and melting curve method. Details including of primer sequences and PCR conditions are listed in Table 4.

**Table 4:** **PCR conditions used in genotyping the inflammatory gene variants**

**Gene**	**rs No**	**Labeled primers**	**Sequence(5'→3')**	**Amplicon (F1/R)/(F2/R) (bp)**	**Annealing temp.(°C)**	**Mg(mM)**	
Tumor necrosis factor-α (TNF-α)	rs1800630	F1(FITC)	ATG GCC CTG TCT TCG TTA AxT G	75/77	62.5	3	
		F2(Texas Red)	GGC CCT GTC TTC GTT AAx GG				
		R(Biotin)	ACA GCA ATG GGT AGG AGA ATG TC				
Interleukin-1A (IL1A)	rs1800587	F1(FITC)	AATAATAGTAACCAGGCAACxTC	91/92	60	2.5	
		F2(Texas Red)	TAATAATAGTAACCAGGCAACxCC				
		R(Biotin)	AGTGGCTAAGTTTGGGAATGG				
Interleukin-1B (IL1B)	rs16944	F1(FITC)	GGTGCTGTTCTCTGCCTxGG	50	60	1.5	
		F2(Texas Red)	GGTGCTGTTCTCTGCCTxAG				
		R(Biotin)	TCAGAGGCTCCTGCAATTGAC				
Interleukin 4 receptor (IL-4R)	rs1801275	F1(FITC)	GCC CCC ACC AGT GGC TAT xGG	49/50	67.5	2.8	
		F2(Texas Red)	GGC CCC CAC CAG TGG CTA TxA G				
		R(Biotin)	CAC CCT GCT CCA CCG CAT GTA				
Interleukin-6 (IL-6)	rs1800796	F1(FITC)	GGC AGT TCT ACA ACA GCx CC	43/42	60	1.5	
		F2(Texas Red)	GCA GTT CTA CAA CAG CxG C				
		R(Biotin)	CTG TGT TCT GGC TCT CCC TG				
Interleukin-10 (IL-10)	rs1800872	F1(FITC)	CAG AGA CTG GCT TCC TAC AxG A	51/53	62.5	2.5	
		F2(Texas Red)	CCA GAG ACT GGC TTC CTA CAx TA				
		R(Biotin)	GCC TGG AAC ACA TCC TGT GA				
Interleukin 1 receptor-associated kinase-1 (IRAK-1)	rs1059702	F1(FITC)	AGGGGCCAGCAAAACGGxAA	99	67.5	2.8	
		F2(Texas Red)	AGGGGCCAGCAAAACGGxGA				
		R(Biotin)	GGGACCCCACCTGATCCAGGC				
Melting curve method							
Gene	rs No	Primers	Sequence(5'→3')	Amplicon (bp)	Annealing temp.(°C)	Mg(mM)	Probe(5'→3')
Tumor necrosis factor receptor superfamily member 1B (TNFRSF1B)	rs1061624	F	CCC TCT GAC CTG CAG GCC AAG	61	65	2	AGC AGA GGC AGC GAG TTG
		R	CCA TGG CAG CAG AGG CTT TCC				
C-reactive protein 2 (CRP2)	rs1130864	F	GAGCTCGTTAACTATGCTGGGA	97	65	3	GCTGGGAAACGGTCCAAA
		R	TTATCTCCAAGATCTGTCCAACTTG				

Genotype distributions of 9 SNPs in participants at first-wave examinations of NILS-LSA before application of exclusion criteria are shown in Table 5. There were no significant differences between genotypic frequencies of our participants and public database samples but in *IL-4R* and *IL-6* (chi-square test, level of significance: 0.05).

**Table 5:** **Genotype distribution of participants at first-wave examinations of NILS-LSA before application of exclusion criteria, and comparison with those of HapMap-JPT or another public database**

**Gene**	**rs No**	**Allele** ^**1)**^	**Genotype N= 2149, Number (%)**			**p-value**
*TNF-α*	rs1800630	A (C)	CC	CA	AA	
Our baseline subjects			1551 (72.2)	431 (20.0)	167 (7.8)	NS
HapMap-JPT			(71.1)	(20.0)	(8.9)	
*TNFRSF1B*	rs1061624	A (G)	GG	GA	AA	
Our baseline subjects			519 (24.1)	1070 (49.8)	560 (26.1)	NS
HapMap-JPT			(29.4)	(48.2)	(22.4)	
*IL1A*	rs1800587	T (C)	CC	CT	TT	
Our baseline subjects			1745 (81.2)	385 (17.9)	19 (0.9)	NS
HapMap-JPT			(76.7)	(20.9)	(2.3)	
*IL1B*	rs16944	T (C)	CC	CT	TT	
Our baseline subjects			600 (27.9)	1096 (51.0)	453 (21.1)	NS
PGA-UW-FHCRC: JPT_GENO_PANEL^2)^			(26.3)	(50.0)	(23.7)	
*IL-4R*	rs1801275	G (A)	AA	AG	GG	
Our baseline subjects			1585 (73.8)	522 (24.3)	42 (1.9)	0.034
HapMap-JPT			(81.4)	(18.6)	(0)	
*IL-6*	rs1800796	G (C)	CC	CG	GG	
Our baseline subjects			1257 (58.5)	762 (35.5)	130 (6.0)	0.007
HapMap-JPT			(75.0)	(22.7)	(2.3)	
*IL-10*	rs1800872	C (A)	AA	AC	CC	
Our baseline subjects			985 (45.8)	938 (43.7)	226 (10.5)	NS
HapMap-JPT			(44.4)	(51.1)	(4.4)	
*IRAK-1*	rs1059702	C (T)	TT	TC	CC	
Our baseline subjects			1444 (67.2)	406 (18.9)	299 (13.9)	NS
HapMap-JPT			(66.3)	(19.8)	(14.0)	
*CRP2*	rs1130864	T (C)	CC	CT	TT	
Our baseline subjects			1889 (87.9)	251 (11.7)	9 (0.4)	NS
HapMap-JPT			(87.2)	(11.6)	(1.2)	

^1)^Reference alleles are indicated in parentheses.

^2)^Variation Discovery Resource. A collaboration between the University of Washington and the Fred Hutchinson Cancer Research Center.

Air-conduction pure-tone thresholds at octave intervals from 0.5 kHz to 8 kHz were measured in a sound-proof booth by trained examiners using a standardized protocol and a diagnostic audiometer (AA-73A and AA-78; Rion, Tokyo, Japan). The pure-tone average threshold level of the better ear at frequencies of 0.5, 1, 2, and 4 kHz (PTABE) was used as an index of hearing status. Hearing impairment was defined as a PTABE greater than 25 dB according to WHO grades [34]. The WHO defines disabling hearing impairment in adults as a permanent unaided PTABE of 41 dB or greater. In view of this, we adopted two hearing impairment criteria for the statistical analyses; PTABE >25 dB and PTABE >40 dB.

### Statistical analyses

Statistical analyses were conducted using the Statistical Analysis System (SAS) version 9.1.3 (SAS Institute, Cary, NC, USA). For univariate analysis, a *t*-test was used to assess differences of continuous variables between two groups, and comparisons of categorical variables were performed using the chi-square test. Fisher’s exact test was also used, as and when the number of cell samples was less than five. Unless otherwise noted, values are expressed as mean ± standard deviation.

Cumulative data were analyzed using GEEs, which take into account the dependency of repeated observations within subjects. GEE is a common statistical model to analyze epidemiological longitudinal data. GEE models were fitted using the GENMOD procedure of Statistical Analysis System version 9.1.3. The GENMOD procedure fits generalized linear models. The correlation structure was specified as autoregressive. We took mode of inheritance in analyses as additive genetic model, which is the prevailing analytical model in genetic epidemiology, assumes that there is a linear gradient in risk with increasing numbers of variant alleles (reference allele homozygotes base). ORs for the additive genetic models of 9 SNPs with the risk of hearing impairment were analyzed. As a combination of moderator variables, three models were analyzed. In model 1, no moderator variables were adjusted; in model 2, age and gender were taken as moderator variables; and in model 3, any history of noise exposure, ear disease, hypertension, diabetes, dyslipidemia, stroke and cardiac disease, and lifetime smoking habit was adjusted in addition to age and gender.

## Abbreviations

PTABE: Better ear pure-tone average
TNF: Tumor necrosis factor
TNFRSF: TNF receptor super family
IL: Interleukin
IRAK1: IL-1 receptor-associated kinase 1
CRP: C reactive protein
CI: Confidence interval
NILS-LSA: The National Institute for Longevity Sciences - Longitudinal Study of Aging
WHO: World Health Organization
SNP: Single-nucleotide polymorphism
GEE: Generalized estimating equation
OR: Odds ratio
CI: Confidence interval
NR2B: N-methyl D-aspartate receptor subunit 2B
5-FU: 5-fluorouracil
CDDP: Cisplatin
SSNHL: Sudden sensorineural hearing loss
PCR: Polymerase chain reaction
SAS: Statistical analysis system
